# Fertility-Preserving Treatment Options in Patients with Malignant Hematological Diseases

**DOI:** 10.5505/tjh.2012.72681

**Published:** 2012-10-05

**Authors:** Mert Küçük, Ali Zahit Bolaman, İrfan Yavaşoğlu, Gürhan Kadıköylü

**Affiliations:** 1 Adnan Menderes University, School of Medicine, Department of Obstetrics and Gynecology, Aydın, Turkey; 2 Adnan Menderes University, School of Medicine, Department of Internal Medicine, Division of Hematology, Aydın, Turkey

**Keywords:** Fertility preservation, Malignant hematological diseases, Ovarian tissue cryopreservation, Embryo cryopreservation

## Abstract

The number of patients of reproductive age diagnosed with various malignant hematological diseases increases every year. These patients undergo chemotherapy, radiotherapy, and various other treatments that may have gonadotoxic effects. The life expectancy of these patients is increasing rapidly due to the variety of treatment options. As such, an increasing number of patients—as well as their parents and spouses—express their concerns about the patient’s fertility post treatment. In the present review it was aimed to provide an overview of current fertility-preserving treatment options and the future of fertility preservation.

## Abstract (tr)

Her yıl üreme çağında çeşitli malin hematolojik hastalıklara yakalanan hasta sayısı artmaktadır. Bu hastalar gonadotoksik etkileri olabilen kemoterapi, radyoterapi ve çeşitli tedaviler almaktadırlar. Hastaların beklenen yaşam süreleri ise çeşitli tedavi seçeneklerinin yardımı ile hızla artmaktadır. Bu nedenle giderek artan sayıda hasta, ebeveynleri ve hastaların eşleri tedavi sonrası hastanın fertilite potansiyeli üzerine endişelerini ifade etmektedirler. Bu derlemede güncel fertilite koruyucu tedavi seçenekleri ve fertilite prezervasyonunun geleceği konusunda bilgi vermek amaçlandı.

## INTRODUCTION

It is well known that some treatment options used in patients with malignant hematological diseases negatively affect fertility. The negative effects of various treatments on fertility, the steadily increasing number of cures available, and improvement in 5-year life expectancy have all served to increase the importance of patient quality of life (QoL). Among patient QoL issues is the desire of patients to become parents post treatment [1]. 

Schover et al. reported that 76% of childless young cancer survivors reported wanting to become a parent and that they were concerned about the effects of cancer treatment on their fertility [1]. Fertility preservation in cancer patients is becoming a more frequent issue in oncological practice. The American Society of Clinical Oncology (ASCO) recently published a clinical guidebook that encourages healthcare professionals to inform patients about and discuss fertility-preserving treatment options [2]. The American Society of Reproductive Medicine (ASRM) Ethics Committee has also expressed its concern for this issue [3]. 

Today, the fertility-preserving treatment options most commonly offered to patients are cryopreservation of sperm and embryos [2,3,4]. Pregnancy has also been made possible via oocyte and ovarian tissue cryopreservation [5]. Other fertility preservation methods are still in the experimental stage. This review aimed to provide an overview of currently used fertility-preserving treatment options, those still undergoing experimentation, and a look into the potential future of fertility preservation. 

In 1948 it was reported that nitrogen mustard had a toxic effect on the testes [6]. Since then, the adverse effects on reproduction of many treatment procedures have become known. 

Chemotherapy can lead to amenorrhea or a reduction in the ovarian reserve [7]. Alkylating agents—particularly cyclophosphamide—are known gonadotoxic agents [7]. Oktay et al. reported that patients treated with alkylating agents had fewer primordial follicles in their ovaries than those that were not treated with these agents [8]. Generally as the dose and duration of treatment increase, so do the negative effects [9]. Younger patients have a larger pool of primordial follicles and are more able to tolerate the chemotherapy. Ovarian follicles are more sensitive to chemotherapy during the proliferative phase of the menstrual cycle [10,11]. Pelvic radiotherapy negatively affects the reproductive system, causing degeneration of primary and primordial follicles [12]. 

In fact, fertility preservation should be guided by two principles: 1. Use of treatments that are the least gonadotoxic as possible; 2. Use of fertility-preserving treatment options when gonadotoxicity cannot be avoided [13]. 

The majority of patients with acute lymphoblastic leukemia (ALL), acute myeloid leukemia (AML), Hodgkin’s lymphoma (HL), and non- Hodgkin’s lymphoma (NHL) have reproductive potential prior to treatment, making treatment-related infertility an important issue. Hematopoietic stem cell transplantation (HSCT) is frequently included in the treatment plan of patients with hematologic diseases. During HSCT gonadotoxic agents known to have a very negative effect on future fertility are used [14]. In fact, the potential effect of each hematological malignancy and each treatment protocol onfuture fertility is unique. What follows is a brief explanationof important malignant hematological disorders andHSCT and fertility preservation options [14]. It is importantto note that gonadotoxic treatments and/or HSCT areused not only in cases of malignancy, but in patients withprecancerous and benign diseases [5].

**Lymphomas**

Among patients with HL and NHL, 5-year survival hasincreased markedly, approaching approximately greaterthan 80%. The success of current treatment modalitiesfor malignant hematologic diseases has facilitated anincrease in clinicians’ ability to focus on such problemsas post-treatment infertility [13]. Semen analysis must beperformed in all male patients with lymphomas prior totreatment. The quality of semen is lower in patients withHL and NHL than in healthy male controls. Among malepatients diagnosed with HL, 21% and 49% had azoospermia,and moderate or mild semen abnormalities, respectively[14]. The exact cause of these abnormalities has notyet been elucidated. These disorders in HL may be relatedto fever or pro-inflammatory cytokines, such as interleukin1 (IL1), IL6, tumor necrosis factor-α, and soluble ILreceptors 2 and 6 [14].

A relationship has not been observed between diseasestage and quality of semen in HL [15,16,17]. The folliclestimulatinghormone level in male patients was suggestedto be a marker of male fertility [18] and inhibin B wassuggested as an indirect marker of male fertility [19].Low levels of inhibin B were associated with impairedspermatogenesis in children and adults receiving chemotherapy[20]. Anti-Müllerian hormone was reported to bean important predictor and marker of gonadal function inwomen that underwent chemotherapy for HL [21].

The most common cause of gonadal dysfunction inpatients with HL is gonadotoxic chemotherapy [15]. Alkylatingchemotherapeutic agents, such as procarbazine and/or cyclophosphamide, cause prolonged azoospermia in90%-100% of men and premature ovarian failure (POF) in5%-25% of younger women under 30 years old; however,the risks are low with radiotherapy alone if pelvic radiationor chemotherapy with alkylating agents is not given[22]. Previously the MOPP (mechlorethamine, vincristine,procarbazine, prednisolone), COPP (cyclophosphamide,vincristine, procarbazine, prednisolone), and MOPP-ABV(alternating cycles of mechlorethamine, vincristine, procarbazine,prednisolone, and doxorubicin, bleomycin,vinblastin, and dacarbazine) regimens were frequently administered, whereas ABVD (doxorubicin, bleomycin, vinblastin, and dacarbazine) and BEACOPP (bleomycin, etoposide, doxorubicin, cyclophosphamide, vincristine, procarbazine, and prednisolone) chemotherapy regimens are currently more popular. 

The MOPP, COPP, and MOPP-ABV chemotherapy regimens are more toxic to the gonads than the other mentioned regimens and may cause secondary leukemia. As such, they are not commonly used to treat HL, as they may have greater gonadotoxic effects in HL patients in whom gonadal damage was evident during the pre-treatment period [22]. The ABVD regimen is a non-alkylating regimen that is less gonadotoxic compared to regimens including alkylating agents. Among those treated with the ABVD regimen, only 33% of male patients experienced transient azoospermia, and 8% of female patients developed POF. In comparison to regimens containing alkylating agents, such as BEACOPP, these rates were significantly lower [18]. The gonadotoxic effects of second-line HL chemotherapy regimens, including DHAP (dexamethasone, cytarabine, and cisplatin), ICE (ifosfamide, carboplatin, and etoposide), and MINE (mesna, ifosfamide, mitoxantrone, etoposide), remain unknown [22]. NHL patients often receive the CHOP regimen or CHOP-based regimens; among such patients, the observed rate of gonadal toxicity is low, both in men and women [23]. 

**Leukemias and HSCT**

Fertility preservation has become an important issue among patients with acute leukemias due to the increase in the success of various chemotherapy protocols. Male leukemia patients had lower pre-treatment semen parameters than healthy controls [24]. Standard chemotherapy regimens used for the treatment of AML and ALL have little toxic effect on reproductive potential [25]. Treatmentrelated infertility in leukemia patients is generally related to HSCT [26]. 

HSCT, along with its associated gonadotoxic conditioning regimens, has been successfully used for the treatment of both lymphoma and leukemia. HSCT may be performed as autologous or allogeneic transplantation. The gonadotoxic effect of HSCT in leukemia patients is related to whether or not a myeloablative or non-myeloablative conditioning regimen is administered, and whether or not total body irradiation (TBI) is used as a conditioning regimen prior to HSCT [27]. Myeloablative pre-transplant conditioning regimens include alkylating agents and/or TBI [28]. Both alkylating agents and TBI are associated with marked germ cell damage and infertility [29]. Azoospermia is more frequently encountered in patients that have undergone allogeneic HSCT and received myeloablative conditioning regimens. Additionally, spermatogenesis is frequently impaired in this group of patients; however, spermatogenesis sometimes improves 9-10 years following gonadotoxic therapy [30]. 

Patients treated with TBI followed by HSCT have a high risk of gonadotoxicity [31]. The gonadotoxic effect of TBI increases in particular when combined with cyclophosphamide [32]. POF is more common when TBI is used for preconditioning. The incidence of POF is lower in cases treated with non-myeloablative conditioning regimens and autologous transplantation [33]. Although rare, recovery of ovarian function can occur years after HSCT in cases in which POF is observed [34]. Radiation can also lead to hypothalamic amenorrhea [35]. One study reported that 68% of patients undergoing radiotherapy of both ovaries developed ovarian failure [36]. Another adverse effect of radiotherapy is a reduction in blood flow to the uterus and a decrease in uterine volume [37]. Pelvic irradiation has been associated with adverse obstetric and neonatal outcomes—among them, spontaneous abortion, low birth weight, and placental anomalies [38,39,40]. Pelvic irradiation has also been associated with placenta accreta and percreta, as well as uterine rupture [39,40,41,42,43,44]. Moreover, patients that undergo allogenic HSCT or irradiation, or patients that develop graft-versus-host disease (GVHD) are reported to be at risk for implantation problems [45]. In addition to gonadal damage, these patients may develop vaginal and cervical stenosis, resulting in deterioration of sexual function, with dyspareunia and reproductive failure, or difficult childbirth [45]. 

Gonadal damage is expected to be extensive in the following patients: patients aged ≥30 years at the time of HSCT, especially those that receive chemotherapy with alkylating agents before HSCT; patients that develop GVHD; patients with a predisposition for infiltration of the gonads; male patients; patients that receive TBI; patients treated with TBI that have undergone allogeneic HSCT; post-pubertal patients [27]. Patients scheduled for HSCT should receive fertility counseling during the pre- and post transplant periods [27]. 

**Fertility Preservation Options **

**Embryo Cryopreservation**

The ASRM Ethics Committee reported that embryo cryopreservation is the most successful method available today for fertility preservation [3]. Embryo cryopreservation consists of the following 4 steps: 1. Controlled ovarian hyperstimulation and induction of multifollicular growth; 2. Retrieval of follicles from the ovaries, generally under transvaginal ultrasound guidance; 3. In vitro fertilization with the partner’s sperm or fertilization via intra-cytoplasmic sperm injection (ICSI); 4. Cryopreservation of the resultant embryos. 

Embryo cryopreservation can be performed via traditional slow-freezing or a rapid freezing method referred to as vitrification. Embryo survival after thawing is expected to be around 90% with the vitrification method, versus 75% with the slow freezing method [46,47]. 

In some of the following cases embryo cryopreservation is unsuitable or difficult to accomplish; 1. The method is not suitable for prepubertal females; 2. In Turkey the patient must be married. Cryopreservation with donor sperm is not legal in Turkey; 3. Supraphysiological estrogen levels during ovarian stimulation are regarded by some clinicians as prohibitive, particularly in patients with hormone-dependent cancers [48]; the use of letrozole, however, is recommended during controlled ovarian hyperstimulation in patients with hormone-dependent cancers [49]; 4. Patients generally need a few weeks to a few months for ovarian stimulation for embryo cryopreservation which sometimes may not be preffered [50]. 

**Sperm Cryopreservation**

Sperm cryopreservation is a fertility preservation method with a high rate of successful outcomes and is easy to implement. The recommendation is that sperm should be collected three times, each after 48 h of abstinence [51]. In cases in which the underlying pathology is HL, testicular cancer, or leukemia the sperm count and/or quality may be low [52]. With the help of supplementary reproduction techniques and, in particular ICSI, success is possible when even a limited number of sperm are frozen and then thawed [53]. Sperm cryopreservation is possible even if chemotherapy or radiotherapy has already been initiated [54]; however, for maintaining DNA integrity in cases of a low sperm count, it is recommended that sperm be collected before such treatment begins. 

Alternatives to obtaining sperm via masturbation include penile vibrator stimulation, testicular aspiration, testicular extraction, electroejaculation [55]. Testicular tissue cryopreservation is another alternative fertility preservation option. Testicular stem cell transplantation and transplantation of frozen-and-thawed testicular cells back to the testes after various gonadotoxic treatments are currently under investigation [13]. 

**Oocyte Cryopreservation**

Oocyte cryopreservation is a method in which—similar to embryo cryopreservation—the ovaries are subjected to controlled hyperstimulation, and then the oocytes are retrieved via a minor surgical procedure [56]. This method is unsuitable for pediatric patients. This method has some advantages and disadvantages in comparison with embryo cryopreservation: 1. Oocyte cryopreservation can be used in women without a partner. 2. Oocytes are much more sensitive to freezing and thawing procedures than embryos [57], and as such the success rate of this method is limited; therefore, ASRM still regards this method as experimental [3]; 3. Following freezing and thawing, thickening of the zona pellucida reduces the chances of fertilization of the oocyte [58]. Although ASRM regards this method as experimental, the technology is rapidly advancing [59]. The increase in oocyte survival rates after freezing and thawing following vitrification is promising, but the rate remains low [60]. In short, oocyte cryopreservation is still in the development stage and research is ongoing; nonetheless, results obtained to date show that that technique has great promise. 

**Ovarian Tissue Cryopreservation **

Ovarian tissue cryopreservation consists of the following: 1. Surgical—and usually—laparoscopic removal of part of the ovarian tissue; 2. Cryopreservation of the removed ovarian tissue; 3. Thawing and processing of the ovarian tissue when the decision to do so is made, after which time the tissue is subjected to heterotopic or orthotopic implantation; in cases of heterotopic implantation the frontal abdominal wall [61] or the forearm [62] is used, whereas orthotopic sites employed are the ovarian fossa and the pelvic peritoneum. Transplantation can also be made to the cortex of the residual ovary [63,64]. 

Ovarian tissue cryopreservation appears to be the only choice for prepubertal females and in particular, for postpubertal patients in whom treatment cannot be postponed, even for a short time (cases in which there is no time for ovarian stimulation either for embryo or oocyte cryopreservation) [65]. Another advantage of ovarian tissue cryopreservation is its applicability during any stage of the menstrual cycle. The resistance of primordial follicles to cryotoxicity compared to that of mature oocytes is another advantage of the method [66]. 

The disadvantages of ovarian tissue cryopreservation are as follows: 1. It requires surgery; 2. It is associated with the risk of thrombosis and hemorrhaging; 3. Although primordial follicles are more resistant, as there no blood flow at the initial ischemia 66% of oocytes losetheir viability [67]; 4. There is a theoretical risk, althoughlow, that with this method malignant cells might find theirway back into the patient’s tissues. The ovaries are seldomthe site of metastasis, but leukemia, neuroblastoma,and breast cancers have been known to metastasize to theovaries [68,69]. This is particularly important in cases ofBRCA1- and BRCA2-positive mutations [70], in which theremoved ovarian tissue must be evaluated histopathologically;5. The rate of success and clinician experience withthis method are quite limited; to date, very few live birthshave been reported as an outcome of this method [71,72].In short, ovarian tissue cryopreservation is a new techniquewith which clinicians have limited experience, andoutcome data are in short supply.

**Ovarian Transposition, and Intensity-Modulated Radiation Therapy (IMRT) and Gonadal Shielding**

The aim of ovarian transposition is to move the ovariesof patients with HL, neuroblastoma, Wilms tumor, andother similar conditions to a location in the body that isoutside the radiation field [71]. The procedure includesthe following: 1. Surgical transposition of the ovaries to anarea outside of the field of radiation; 2. Following radiotherapy,the ovaries are returned to their original location.The success rate for this procedure is reported to be 16%-90% [73]. Ovarian transposition can be performed priorto irradiation via laparotomy or laparoscopy [74]. Applicationof metal clips to the ovaries during the procedure isuseful in guiding subsequent X-ray localization. Additionally,a sample of ovarian tissue can be excised during theprocedure for cryopreservation. The risks associated withovarian transposition are postoperative chronic pelvic painand pelvic adhesions. Although rare, it should also be keptin mind that the ovaries may migrate back to their formerlocations [75]. Moreover, damage to the ovaries resultingfrom the combination of chemotherapy and radiotherapycannot be prevented with this method.

IMRT is a relatively new radiation technique that facilitatesdelivery of radiation to multiple targets while sparingadjacent tissues. This method is used to minimize the distributionof radiation and the harmful effects of radiationon the ovaries and uterus [76].

Shielding the uterus and ovaries to as great a degreeas possible during radiotherapy or dividing the TBI dosesmay be of benefit to avoid gonadotoxicity [65].

**Ovarian Suppression with a Gonadotropin-Releasing Hormone (GnRH) Analog**

It is known that the ovaries in prepubertal females are more resistant to chemotherapy compared to postpubertal females. GnRH analog treatment is thought to convert the hormonal environment in post-pubertal females to the prepubertal environment, so that the ovaries become more resistant to the toxic effects of chemotherapy. A meta-analysis reported that the use of GnRH analogs prior to chemotherapy was an effective method [77]. Nonetheless, to date the effectiveness of GnRH analog treatment for fertility preservation remains inconclusive [8] and additional large-scale randomized studies are necessary. 

**Xenotransplantation **

Xenotransplantation—transplantation of human ovarian tissue into other species—is in the experimental stage [78,79]. Xenotransplantation has been used successfully to create functional oocytes [80]. As xenotransplantation is still in the experimental stage, the procedure raises issues of safety and ethics. The transfer of non-human DNA or contamination with viruses may turn out to be risks associated this procedure [81]. 

**Stem Cells**

Stem cells are able to differentiate into diverse specialized cell types. Research continues on methods of cloning germ cells from adult somatic cells and the creation of new differentiated cells from stem cells [82,83]. 

**Current Legislation in Turkey**

According to Turkish regulations concerning assisted reproductive treatment methods and assisted reproductive treatment centers, partners that undergo such treatment may only receive their own reproductive cells. It is illegal to make use of a donor in any way—to harvest an embryo from a donor, transfer an embryo from one assisted reproductive treatment candidate to another via use of eggs or sperm harvested from a candidate, and to use or implant embryos in assisted reproductive treatment candidates that have been harvested from non-candidates [84]. 

When embryos have been retrieved, these can be cryopreserved with the consent of both partners. If an embryo has been preserved over one year, each year the couple must confirm their desire to continue the preservation by submitting a signed petition. Upon the consent of both partners, in the event of the death of one of the partners or a legally established divorce, or at the end of the determined period, a record is kept by a commission to be appointed by the health directorate of the city and the embryos are destroyed [84]. 

It is legal for both men and women to preserve reproductive cells and gonad tissue before undergoing treat ment that may damage gonad cells, such as chemotherapy or radiotherapy. To maintain the security of the materials, reproductive cells and gonad tissue are preserved together with a DNA analysis of the donor. In the event that preservation exceeds one year individuals must apply to the authorities with a signed petition confirming the desire to continue the preservation of the tissues/cells. Anytime an individual fails to renew the protocol, or upon his/her request, or in the event of death, a record is kept by a commission to be appointed by the health directorate of the city and the cryopreserved reproductive cells and gonad tissues are destroyed [84]. Reproductive cells and gonad tissues, as well as frozen embryos, may be preserved for a maximum of five years; preservation exceeding five years is subject to the approval of the Ministry of Health [84]. 

**Fertility Preservation Risks and Ethical Considerations**

Although fertility preservation treatment has undergone significant advancement and continues to generate great interest, and despite the fact that many oncology patients and their families currently seek such treatment, the ethical issues surrounding its use remain unresolved. At the same time, promising new developments and methods of fertility preservation (e.g. xenotransplantation) complicate the ethics of such treatment. In addition, as each individual and culture has unique psychosocial, cultural, and religious sensitivities and realities, the ethical issues associated with fertility preserving treatment vary accordingly. 

An important ethical issue concerns non-adult patients and the informed consent process; in particular, is it ethical for such decisions to be made by the parents of minors. As such, scientific authorities have stressed the importance of ensuring that children and adolescents are involved in the informed consent process to a degree that is age appropriate [85]. When children or adolescents are concerned, besides age, having the capacity to understand the situation in which the individual is in is more important not age but when participating in the fertility preservation process. It is, however, not always easy to determine whether such capacity exists. A family’s decision about a minor’s future reproductive choice can, for example, result in the rejection of cryopreservation, which in later years becomes an ethical issue if the adult child now wished to become a parent. Additionally, parents that opt for gamete cryopreservation for their child can have the cells destroyed before the patient has reached legal age. Still, another ethical issue concerns the future of cryopreserved embryos or gametes in the event a patient dies, i.e. a healthy partner wants to become pregnant with a preserved embryo after her partner has died. 

An important ethical consideration is the degree to which patients and their families are informed about experimental fertility preservation options. The information provided to patients should specify that the process is experimental and objective data provided should include information on success rates. Incorrectly raising the hopes of patients and their families when experimental methods are being tested will inevitably bring about many problems. In addition, all oncology patients must be informed about fertility preservation options and their written informed consent must be obtained prior to such treatment. If patients or their legal guardians refuse fertility preservation, clinicians must document their choice. When a legal issue arises and patients claim that they were not informed about fertility preservation options, or if they admit that they were, but claim to have actually asked for one of the options, in order to resolve a possible dispute, there needs to be a written consent that was signed by the patient and/or the legal guardian before the treatment for cancer. Another issue is the matter of who should obtain a patient’s informed consent—the patient’s attending physician or the reproductive endocrinologist. 

It is a possibility that malignant cells may be reintroduced when tissue is re-transplanted during the ovarian tissue cryopreservation procedure [86]. The time needed for oocyte or embryo cryopreservation and sometimes the clinician’s feeling pressed enough to act quickly to start the treatment for cancer or the necessity to do so can also be problematic [87]. The risk of transferring non-human DNA or viruses to humans when employing xenotransplantation must always be a consideration [81], as well as the potential hazards of the interaction of human and animal DNA [88]. Another important ethical issue concerns problems that might arise if a patient becomes pregnant following fertility preservation treatment and then has oncological relapse during the pregnancy [87]. 

Although there are no studies in this area, it can be assumed that clinicians’ awareness of the matter of fertility preservation may be lacking. The clinicians report that presentation of oncology patients to fertility preservation clinics is not a frequent occurrence. As such, we think that these methods are either underestimated or undervalued by such patients. Hematologists should continue to search objectively for the underlying problems and the reasons for this probable underestimation.

## CONCLUSION

Sperm and embryo cryopreservation are fertility preservation methods proven to be successful. Oocyte cryopreservation and ovarian tissue cryopreservation have also resulted in successful births. Although xenotransplantation, stem cell utilization, and other experimental methods are still under development, they have potential for future success. 

**Conflict of Interest Statement **

The authors of this paper have no conflicts of interest, including specific financial interests, relationships, and/ or affiliations relevant to the subject matter or materials included.
